# *AUTS2* Gene: Keys to Understanding the Pathogenesis of Neurodevelopmental Disorders

**DOI:** 10.3390/cells11010011

**Published:** 2021-12-21

**Authors:** Kei Hori, Kazumi Shimaoka, Mikio Hoshino

**Affiliations:** Department of Biochemistry and Cellular Biology, National Institute of Neuroscience, NCNP, 4-1-1 Ogawahigashi, Kodaira, Tokyo 187-8502, Japan; kshimaoka@ncnp.go.jp

**Keywords:** autism susceptibility candidate 2 (AUTS2), autism spectrum disorders (ASD), epigenetic modulation, cytoskeleton, neurogenesis, neuronal migration, neuritogenesis, synapse, cerebellum

## Abstract

Neurodevelopmental disorders (NDDs), including autism spectrum disorders (ASD) and intellectual disability (ID), are a large group of neuropsychiatric illnesses that occur during early brain development, resulting in a broad spectrum of syndromes affecting cognition, sociability, and sensory and motor functions. Despite progress in the discovery of various genetic risk factors thanks to the development of novel genomics technologies, the precise pathological mechanisms underlying the onset of NDDs remain elusive owing to the profound genetic and phenotypic heterogeneity of these conditions. Autism susceptibility candidate 2 (*AUTS2*) has emerged as a crucial gene associated with a wide range of neuropsychological disorders, such as ASD, ID, schizophrenia, and epilepsy. AUTS2 has been shown to be involved in multiple neurodevelopmental processes; in cell nuclei, it acts as a key transcriptional regulator in neurodevelopment, whereas in the cytoplasm, it participates in cerebral corticogenesis, including neuronal migration and neuritogenesis, through the control of cytoskeletal rearrangements. Postnatally, AUTS2 regulates the number of excitatory synapses to maintain the balance between excitation and inhibition in neural circuits. In this review, we summarize the knowledge regarding AUTS2, including its molecular and cellular functions in neurodevelopment, its genetics, and its role in behaviors.

## 1. Introduction

The disruption of brain development has been implicated in various psychiatric and neurological illnesses with neurodevelopmental origins. The underlying pathological processes of these diseases often begin early during fetal development and continue into late brain maturation, resulting in pervasive and long-lasting abnormalities in brain structure and function. Neurodevelopmental disorders (NDDs) are a broad spectrum of early onset syndromes affecting cognition, memory, speech, emotions, and behaviors, with a prevalence in children exceeding 3% worldwide [1,2]. Typically, NDDs encompasses a wide range of neuropsychiatric illnesses, including intellectual disability (ID), autism spectrum disorders (ASD), attention-deficit hyperactivity disorder (ADHD), schizophrenia, cerebral palsy, epilepsy, and bipolar disorder [3,4]. In the past decade, the rapid advancement of genomics technologies has led to the identification of a number of gene mutations as well as chromosomal structural variants involved in NDDs [5,6]. Moreover, systems biological analyses have pointed out that a significant number of the genes responsible for NDDs, especially ASD, can converge into neurodevelopmental pathways such as chromatin remodeling, transcriptional regulation, and synaptic function [1,7,8,9,10]. Thus, the understanding of the molecular pathological mechanisms underlying NDDs has progressed; however, due to the substantial genetic and phenotypic heterogeneity of these disorders, the precise etiology of NDDs remains unclear.

The autism susceptibility candidate 2 (*AUTS2*, also termed “activator of transcription and developmental regulator” by the HUGO Gene Nomenclature Committee, #14262) gene was initially reported as an ASD risk gene disrupted by de novo balanced chromosomal translocation in monozygotic twins with ASD in 2002 [11]. Since then, various genomic structural variants, such as deletions, duplications, inversions, and translocations, as well as single nucleotide polymorphisms (SNPs) in the *AUTS2* locus have been associated not only with ASD but also a variety of other psychiatric and neurological illnesses, such as ID, schizophrenia, ADHD, language disorder, epilepsy, depression, and drug dependence [12,13,14,15,16,17,18,19,20,21]. Additionally, patients with *AUTS2* mutations often display other pathological conditions including developmental delay, microcephaly, short stature, and craniofacial dysmorphisms; a series of such symptoms are termed “*AUTS2* syndrome” [14,22]. In this review, we summarize the knowledge of the molecular and cellular functions of *AUTS2* in neurodevelopment and the involvement of this gene in NDDs.

## 2. Structure and Expression of the *AUTS2* Gene

**Figure 1:**
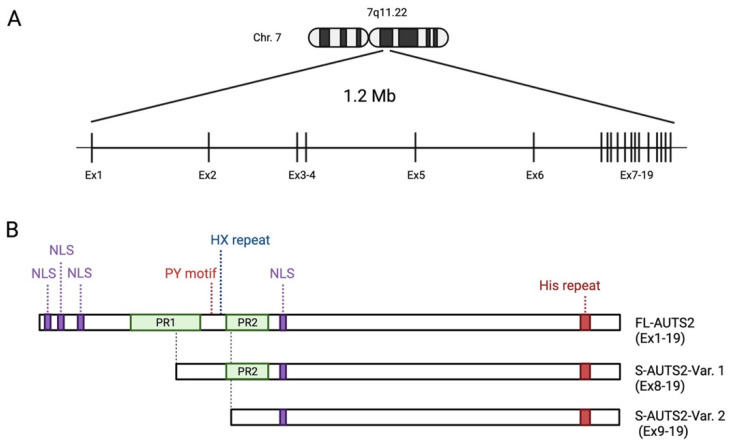
Schematic showing the human *AUTS2* gene genomic region and the structure of AUTS2 protein isoforms: (**A**) Genomic structure of human *AUTS2* locus on chromosome 7q11.2 (Chr. 7), consisting of 19 exons (Ex). (**B**) Schematic representation of the protein structure of full-length and C-terminal short AUTS2 isoforms. The location of predicted domains, motifs, and other characteristic sequences are shown. NLS: nuclear localization signal sequence; PR: proline-rich domains. Illustration created with BioRender.com.

*AUTS2* is a large gene spanning 1.2 M bases on human chromosome 7q11.22 (Figure 1A) [12,23]. It consists of 19 exons, the first 6 of which are separated by long introns at the 5′ end, whilst the remaining 13 are compact with clustered smaller introns at the 3′ end. The full-length *AUTS2* transcript encodes a protein with 1259 amino acids (aa) in humans (NM_015570) and 1261 aa in mice (NM_177047), although various isoforms are generated by alternative splicing and multiple transcription start sites (TSS). In the human brain, a short 3′ *AUTS2* transcript generated from TSS within exon 9 has been identified by 5′ RACE (5′ rapid amplification of cDNA ends) [14]. In the developing mouse brain, two short C-terminal AUTS2 isoforms translated from exon 8 (S-AUTS2-Var1; ~88 kDa) and exon 9 (S-AUTS2-Var2; ~78 kDa), as well as the full-length AUTS2 (FL-AUTS2; ~170 kDa) were detected by 5′ RACE and immunoblot experiments [24,25]. Additional alternative splicing *AUTS2* variants skipping exons 10 and 12 in humans (corresponding to exons 11 and 13 in mice) were also found in the fetal brain [26].

In the mouse cerebral cortex, FL-AUTS2 and S-AUTS2-Var1 appear from the early embryonic stages [24,27]. The long isoform shows the highest expression level at later embryonic stages around embryonic days 16–18, and is also present postnatally; although, it gradually decreases thereafter. In contrast, the short isoform is transiently expressed in embryonic brains and disappear postnatally. Interestingly, in mouse embryonic stem cells (mESCs), an isoform switch from the long to short AUTS2 isoform occurs during the transition from undifferentiated to differentiated cortical neuronal states [26].

In the normal mouse brain, S-AUTS2-Var2 is barely detectable compared to other AUTS2 isoforms. In contrast, the expression of this shortest isoform was abnormally increased in mutant mice lacking exon 8 of *Auts2* [24,25]. These findings suggest that there are internal enhancer/promoter regions for the alternative transcription of S-AUTS2, tightly regulating the temporal expression pattern of AUTS2 isoforms in the brain. Several potential enhancer regions have been identified within the introns of this gene in zebrafish and mice [12]. Furthermore, Kondrychyn et al. demonstrated that the *AUTS2* ortholog in zebrafish, *auts2*, potentially has 13 unique TSSs, and more than 20 alternative transcripts were found to be produced from this gene locus [28]. Therefore, the complexity of this gene may be substantially higher in mammals as compared to other species than previously identified. In humans, some of the genomic variants were identified within intronic regions of the *AUTS2* locus in patients with NDDs, implying that disorganized expression of AUTS2 may be associated with the onset of these disorders [21]. *Auts2* expression is reportedly regulated by T-box brain protein 1 (Tbr1), a transcription involved in the development of cortical deep-layer projection neurons and also implicated in ASD [29,30]. In addition, methyl CpG binding protein 2 (MECP2), whose gene mutations are associated with Rett syndrome and ASD, suppresses *AUTS2* expression [31,32].

*Auts2* is widely expressed in multiple regions of the developing mouse brain, but particularly strong levels are observed within areas related to higher cognitive functions, such as the cerebral cortex, the Ammon’s horn and dentate gyrus of the hippocampus, and the cerebellum [33]. In postnatal and fully developed mouse brains, *Auts2* expression is restricted to a few types of neurons, but is strong in glutamatergic projection neurons in the prefrontal cortex, pyramidal CA neurons in the hippocampus, granule neurons of the dentate gyrus, and cerebellar Purkinje and Golgi cells [33,34].

## 3. Genotype–Phenotype Correlations of *AUTS2* Syndrome

As noted, a wide range of neurodevelopmental phenotypes in the patients with *AUTS2* syndrome have been reported in individual studies; however, few studies have systematically reviewed the relationship between genotype and phenotype using a relatively large cohort of patients with *AUTS2* mutations. In 2013, Beunders et al. collected the clinical data of patients (17 individuals and 4 family members) carrying exonic *AUTS2* deletions from a large cohort (49,684 samples) with ID and/or multiple congenital anomalies, and examined the genotype–phenotype correlations in *AUTS2* syndrome [14]. In that study, they originally established an *AUTS2* syndrome severity score (ASSS) system, which was based on the sum of 32 clinical features observed with a frequency of over 10% in the first cohort of individuals with *AUTS2* mutations. The ASSS system includes physical (growth, feeding, dysmorphic features, and skeletal disorders) and neuropsychiatric (neurological and neurodevelopmental disorders) phenotypes and congenital anomalies. Their paradigm revealed that the patients with deletions of the conserved C-terminal regions of AUTS2 (exons at the 3′ of the *AUTS2* gene) showed severe *AUTS2* syndrome phenotypes including neurodevelopmental disorders and dysmorphic features. In contrast, the individuals with small in-frame deletions of the different combinations of exons 2–5 at a non-conserved 5′ region (N-terminus of AUTS2) displayed lower ASSS values and milder pathological features [14].

After the initial description by Beunders et al., a meta-analysis using the data of 31 patients with *AUTS2* syndrome obtained from seven studies, in addition to five newly identified clinical cases, supported that the patients with ADHD and/or ASDs, who carry mutations at 3′ end of the *AUTS2* locus showed higher ASSS values compared with individuals with *AUTS2* aberrations at the 5′ end of the locus [35]. These findings imply that the ablation of C-terminal region of AUTS2 may contribute to the onset of *AUTS2* syndrome. Meanwhile, the individuals carrying a frame shift microdeletion within exon 6 or 7, which would only disrupt full-length *AUTS2* transcript but were unlikely to affect the C-terminal *AUTS2* short transcripts [36], have reportedly exhibited severe *AUTS2* syndrome phenotypes. Taken together, this evidence implies that the deletion of the C-terminal region of full-length *AUTS2* transcript and/or the loss of expression of C-terminal *AUTS2* short transcripts may contribute to the onset of *AUTS2* syndrome.

## 4. Protein Structure of AUTS2

Protein primary structure analysis predicts that AUTS2 possesses several putative nuclear localization signal sequences and two proline-rich domains (PR1 and PR2) (Figure 1B) [11,21]. Other predicted protein motifs include a PY (Pro-Tyr) motif (PPPY), eight CAG (His) repeats, several protein kinase phosphorylation sites, and putative SH2- and SH3-binding domains; however, the exact role of these motifs has not been well analyzed. The PY motif could act as a binding motif to a WW domain that is present in the activation domain of several transcription factors [37]. The His repeat is found in some nuclear proteins, which have been reported to function in the targeting of proteins to subnuclear compartments, such as nuclear speckles [38]. AUTS2 is also predicted to contain several conserved domains that may be involved in RNA metabolism. Castanza et al. found that AUTS2 binds to RNA transcripts related to various biological processes [39]. Proteomic analyses identified several RNA-binding proteins (RBPs) as the AUTS2-binding molecules in the mouse neonatal cerebral cortex, such as RNA splicing factors (splicing factor proline-glutamine rich (SFPQ), serine-rich splicing factor 3 (SRSF3)), the RNA helicases (DDX5 and 17), and the RBP, NONO [39]. Although the physiological significance of the interaction of AUTS2 with RNAs or RBPs remains to be clarified, it is conceivable that AUTS2 may control protein expression through RNA metabolism in addition to transcriptional regulation, as described below.

AUTS2 has an additional characteristic histidine-rich sequence (HX repeats) between the PR domains. Individuals carrying de novo in-frame microdeletion within the region corresponding to HX repeats in the *AUTS2* locus display NDDs, including cognitive delay, microcephaly, and craniofacial dysmorphisms [40,41]. Liu et al. recently found that AUTS2 interacts with a histone acetyltransferase P300/CBP via its HX repeats motif, which is required for the differentiation of neural progenitor cells into neurons [41].

## 5. Cytoplasmic AUTS2 Functions in Cytoskeletal Organization

**Figure 2:**
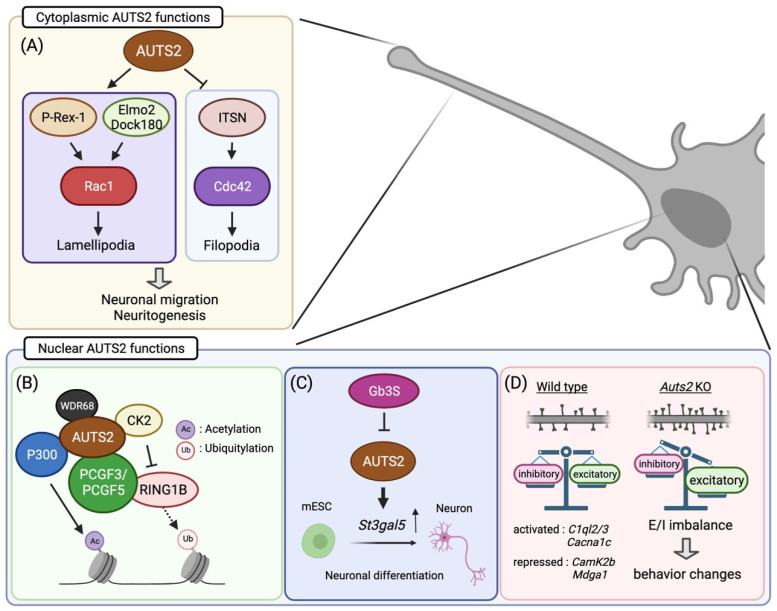
Summary of the molecular and cellular functions of AUTS2 in neurodevelopment: (**A**) Cytoplasmic AUTS2 activates the Rac1 signaling pathway via P-Rex1 and Elmo2/Dock180 complex, promoting neuronal migration and neurite extension. In contrast, AUTS2 inhibits Cdc42 activities to suppress filopodia formation. (**B**–**D**) In cell nuclei, AUTS2 activates gene transcription through histone modification by interacting with the PRC1 complex (**B**). AUTS2 represses the monoubiquitylation activity of RING1B by recruiting CK2 while promoting histone acetylation via P300. (**C**) AUTS2 controls neuronal differentiation from mouse embryonic stem cells (mESCs) through transcriptional activation of the sphingolipid-producing enzyme gene, *St3gal5*. In mESC, AUTS2 expression is repressed by globo-series glycosphingolipids generated from Gb3 synthase, Gb3S. (**D**) AUTS2 modulates the E/I balance by limiting the number of excitatory synapses. Loss of *Auts2* leads to increased dendritic spine formation, disturbing the E/I balance within neural circuits. AUTS2 regulates the expression of the gene related to synapse development and functions. Moreover, *Auts2* mutant mice display behavioral abnormalities in social interaction, vocal communication, cognition, and motor skills. Illustration created with BioRender.com.

AUTS2 is found in the cell nuclei of glutamatergic neurons in the mouse cerebral cortex during early developmental stages [24]. As neural development proceeds, it also appears in the cytoplasm of differentiated neurons, including dendrites, axons, and cell bodies. Subcellular localization analyses showed that FL-AUTS2 was localized in both the nucleus and cytoplasm, whereas the S-AUTS2 isoforms were exclusively nuclear [24]. Cytoplasmic AUTS2 is involved in cell motility and morphogenesis by regulating reorganization of the actin cytoskeleton (Figure 2A) [24]. AUTS2 activates the Rho-family small GTPase Rac1 via guanine exchange factors (GEFs), such as P-Rex1 or the Elmo2/Dock180 complex, leading to lamellipodia formation in neuroblastoma cell lines. In contrast, AUTS2 inhibits filopodia formation by suppressing the activities of another Rho-family G protein, Cdc42, through interaction with its GEF, intersectin 1 and 2.

## 6. Transcriptional Regulation by Nuclear AUTS2

Nuclear AUTS2 functions as a regulator of gene transcription during neurodevelopment. Genomic profiling of AUTS2 by chromatin immunoprecipitation sequencing, in combination with transcriptome analysis (RNA sequencing), revealed that it interacts with the promoter and enhancer regions of the genes related to brain development as well as those associated with NDDs, many of which are actively expressed in the developing mouse forebrain [42]. In addition, AUTS2 shares DNA-binding motifs with some transcription factors that are involved in neural development, including TCF3, Pitx3, and FOXO3, implying that AUTS2 functions in transcriptional activation [42]. Gao et al. found that AUTS2 interacts with an epigenetic regulator type I Polycomb repressive complex (PRC) 1 (Figure 2B) [27]. PRC1 canonically functions as a transcriptional repressor to maintain gene silencing through the compaction of local chromatin by depositing monoubiquitylation of histone H2A at lysine-119 (H2AK119ub1) via its core component RING1A/B [43]. Incorporation of AUTS2 into the PRC1 complex converts this complex to a transcriptional activator by recruiting two other components, casein kinase 2 (CK2), and histone acetyltransferase P300/CBP [27]. CK2 inhibits the monoubiquitylation activity of RING1 proteins, while P300 promotes transcription by acetylating histone H3 at lysine-27 (H3K27ac). In mESCs, the WD40-repeats-containing protein WDR68 mediates transcriptional activation by induction of the AUTS2-PRC1 complex [44]. On the other hand, Monderer-Rothkoff et al. demonstrated that in the luciferase reporter assay, while both FL-AUTS2 and S-AUTS2 activated gene transcription in non-neuronal HEK293 cells, FL-AUTS2 or N-terminal AUTS2 fragment (encoded by exons 1–9) can function as transcriptional repressors in neuroblastoma Neuro2A cells [26]. Whether AUTS2 acts as a transcriptional activator or repressor may therefore depend on the intracellular context, that is, which AUTS2-binding co-factor(s) are expressed.

## 7. The Role of AUTS2 in Neurogenesis

Since the first report by Sultana et al. in 2002, a number of clinical genomic analyses have demonstrated the association of *AUTS2* mutations with NDDs, albeit the molecular and biological functions of this gene have long remained unknown. In 2013, two different groups attempted to determine the function of AUTS2 by means of knockdown experiments using morpholino in a zebrafish model [12,14]. The *auts2* morphants displayed developmental abnormalities, including microcephaly and craniofacial dysmorphisms (smaller jaw size). In the morphant brains, neuronal cells were markedly decreased in various regions, such as the cerebellum, optic tectum, and retina, which was likely based on an increase in apoptosis and/or a decrease in neuronal differentiation [12,14]. Co-introduction of full-length human *AUTS2* mRNA together with the *auts2* morpholino restored morphant phenotypes back to normal. Moreover, the 3′-end short transcript corresponding to a human AUTS2 short isoform could also rescue the phenotype, suggesting that the C-terminal region of AUTS2 contains the domains crucially involved in neural development. This is likely correlated with the observation that the severity of the symptoms of *AUTS2* syndrome is prominent in individuals with disruption at the 3′ end of the *AUTS2* locus [14]. Mice lacking *Auts2* exhibit hypoplasia in the dentate gyrus as well as in the cerebellum [34,39,45]. These findings suggest that AUTS2 plays a key role in neural development in the brain.

In addition, the in vitro analyses using mESCs demonstrated that AUTS2 is involved in neuronal differentiation through histone modifications together with the aforementioned PRC1 complex, including P300, CK2, and WDR68 [41,44,46]. Similar to the findings in the zebrafish model, disrupting the *Auts2* gene in mESC led to increased cell death during differentiation into neurons, which was rescued by expressing the C-terminal AUTS2 shot isoform as well as FL-AUTS2 [26]. Meanwhile, forced expression of FL-AUTS2 in mESCs leads to the delay of neuronal differentiation [34]. Furthermore, Russo et al. found that AUTS2 promotes neuronal differentiation through transcriptional activation of the sphingolipid-modifying enzyme GM3S (Figure 2C) [46]. Taken together, these findings suggest that AUT2 controls neuronal differentiation by transcriptional regulation via histone modification, an epigenetic mechanism.

## 8. AUTS2 Regulates Neuronal Migration and Neuritogenesis during Corticogenesis

In primary cultured hippocampal neurons, forced expression of FL-AUTS2 promotes neurite outgrowth, whereas co-expression of dominant-negative forms of Rac1 or Elmo2 blocks this effect [24]. Furthermore, loss of *Auts2* results in an impairment of axonal elongation of cortical commissural neurons in the developing mouse cerebral cortex, which is restored by expression of wild-type Rac1 in *Auts2*-deficient neurons; this suggests that the AUTS2-Rac1 signaling pathway plays an important role in the neuritogenesis of cortical neurons (Figure 2A) [24].

The AUTS2-Rac1 pathway is also required for neuronal migration (Figure 2A). Disruption of *Auts2* leads to a delay in neuronal migration in the developing mouse cerebral cortex [24]. In *Auts2*-deficient migrating neurons, the activity of c-Jun N-terminal kinase, which is a Rac1 downstream target, is markedly decreased. However, the observed defects in cell migration were restored by the introduction of FL-AUTS2 or wild-type Rac1, but not a short isoform of AUTS2 [24]. In addition, nuclear export sequence (NES)-tagged FL-AUTS2 also rescued the defects in migration, suggesting that cytosolic FL-AUTS2 functions in cortical neuronal migration and subsequent neurite formation by regulating cytoskeletal rearrangements through activation of the Rac1 signaling pathway.

Neuronal migration and subsequent neurite formation are important initial steps in fetal neurodevelopmental processes to properly assemble cortical structures as well as functional neural circuits. Dysregulation of cortical neuronal migration has been associated with NDDs, including ASD [47]. In humans, individuals with *AUTS2* mutations exhibit a wide range of pathological features, including microcephaly, corpus callosum hypoplasia, and psychiatric illnesses. Therefore, defects in neuronal migration and neuritogenesis due to loss of AUTS2 function in the cytoplasm may partly reflect the complex phenotypes of *AUTS2* syndrome.

## 9. AUTS2 Restricts the Number of Excitatory Synapses to Regulate the E/I Balance

Synaptogenesis is a critical neurodevelopmental process for the establishment of the basis underlying higher cognitive brain functions. During postnatal development and in adulthood, the formation and maintenance of an appropriate ratio of excitatory and inhibitory synapses ensures proper excitation/inhibition (E/I) balance in neural circuits [48,49,50]. In contrast, a disturbed E/I balance within the neural circuitry is thought to be associated with NDDs, including ASD, ADHD, schizophrenia, and epilepsy [51,52].

AUTS2 modulates E/I balance by restricting the number of excitatory synapses, suppressing newly formed dendritic spines, and/or promoting the pruning of existing spines during postnatal brain development (Figure 2D) [25]. Loss of *Auts2* leads to increased excitatory synapse formation in pyramidal neurons in multiple forebrain areas, including the medial prefrontal cortex (mPFC), auditory cortex, and hippocampus in the mouse brain as well as primary cultured mouse hippocampal neurons [25]. Interestingly, targeted disruption of *Auts2* in forebrain neurons during late postnatal stages results in excess spine formation, suggesting the involvement of AUTS2 in the regulation of synaptic homeostasis in mature brains. In contrast, AUTS2 does not affect the number of inhibitory synapses. In the *Auts2* mutant hippocampus, excitatory synaptic inputs were increased, while inhibitory synaptic inputs were not altered, resulting in increased excitability in local neural circuits [25]. The aberrant excitatory synapse formation in *Auts2*-deficient neurons is rescued by re-expression of FL-AUTS2 but not S-AUTS2 isoforms. Moreover, the cytoplasmic form of NES-FL-AUTS2 does not restore the synaptic phenotype. Therefore, it is suggested that AUTS2 can restrict the number of excitatory synapses by regulating the transcription of genes related to synapse development by acting as a component of PRC1 or by other regulatory mechanisms. Transcriptome analyses revealed several candidate AUTS2 downstream target genes related to synapse formation and/or functions such as *Mdga1*, *Reln*, *Camk2b*, *Cacna1c*, and *C1ql2/3* [25]. However, it has not been clarified which of these genes are directly regulated by AUTS2 and responsible for the synaptic pathogenesis caused by *Auts2* mutations.

## 10. AUTS2 Is Involved in Cerebellar Development

The cerebellum has traditionally been thought to function in voluntary motor coordination, but accumulating evidence suggests that it is associated with higher-order brain functions, such as social cognition, the reward system, and emotional processing [53,54,55,56]. Among neurons in the cerebellar cortex, Purkinje cells (PCs) play a pivotal role as the sole output neurons to transmit various information processed in the cerebellum to higher brain regions [57,58,59].

*Auts2* is expressed in the cerebellar primordium during early prenatal developmental stages [33]. In adult stages, the expression of AUTS2 is confined to PCs and Golgi cells in the granule cell (GC) layer in the cerebellar cortices [34]. Loss of *Auts2* was shown to result in reduced cerebellar size in mice [34]. In *Auts2* cerebellar conditional knockout (cKO) mice, the number of PCs and GCs is diminished [34]. Coincidently, some patients with *AUTS2* mutations exhibit smaller cerebella [40,41]. Given that AUTS2 is expressed in PCs but not GCs, AUTS2 may directly regulate PC neurogenesis. It is also known that PCs promote the amplification of GC progenitors by secreting sonic hedgehog (SHH), eventually contributing to cerebellar size expansion [60]. Therefore, in *Auts2* cKO mice, GCs might fail to proliferate because of the decrease in the overall amount of SHH along with the reduction in PC numbers, thus causing a reduction in cerebellar size.

The PCs in *Auts2* cKO cerebella show impaired dendrite maturation, including a delay in the regression of surplus dendrites from the cell soma during the early postnatal stages and subsequent dendrite outgrowth, resulting in diminished molecular layer thickness [34]. How exactly AUTS2 promotes the maturation of PC dendrites is unknown; however, since the Rac signaling pathway has been reportedly involved in the dendrite morphogenesis of PCs [61], it is possible that cytoplasmic AUTS2 can be directly involved in these processes via cytoskeletal rearrangements through Rac1 activation, as observed in cerebral cortical neurons. Alternatively, nuclear AUTS2 may control the maturation of PC dendrites by regulating the expression of genes involved in dendrite development.

In the postnatal stages, PCs receive excitatory presynaptic inputs from a single climbing fiber (CF) innervated from the inferior olive nucleus neurons outside the cerebellum, in addition to accepting other excitatory inputs from multiple parallel fibers (PFs) extended from GCs [62]. During the early postnatal period, multiple CFs project to each PC cell body. Subsequently, a single CF with the strongest excitatory transmission is selected to translocate along the primary PC dendrites to form CF synapses, while the remaining CFs are removed. *Auts2* cKO mice show impairments in CF synapse translocation during development, resulting in a decreased number of CF synapses in adulthood [34]. Moreover, electrophysiological experiments revealed that the majority of *Auts2* cKO PCs still received multiple CF inputs even in adulthood, suggesting that the excess CF elimination process is impaired by loss of *Auts2* [34].

In contrast, AUTS2 restricts the number of PF synapses in PCs. Loss of *Auts2* leads to increased PF synapse formation [34]. Furthermore, *Auts2*-deficient PCs exhibit enhanced excitatory neurotransmission in PF synapses. No such alterations were observed in inhibitory synapses. This is evidence implying that the E/I balance in PCs could be disturbed in *Auts2* cKO mice. Moreover, *Auts2*-deficient PCs show reduced expression of the P/Q-type voltage-dependent Ca^2+^ channel gene *Cacna1a*, which has been reported to regulate excitatory synapse development, including CF synapse elimination and PF synapse boundary formation [63,64,65]. Therefore, it is possible that AUTS2 can regulate excitatory synapse development in PCs through transcriptional regulation of the expression of relevant genes with the same mechanism as observed in telencephalon [25].

Emerging evidence has pointed out a link between cerebellar dysfunction and NDDs, such as ASD, and studies in both human and animal models have shown a decrease in the number or disrupted function of PCs [66,67]. AUTS2 is a key regulator involved in a wide range of processes during PC development, including neurogenesis, dendrite maturation, and PC-centric neural circuit assembly. However, the molecular mechanisms underlying the regulation of AUTS2 in PC development are not well understood. In addition, the function of this molecule in other AUTS2-expressing cells, such as Golgi cells, has not been determined, and how *Auts2* loss affects the development or function of these cells remains to be clarified.

## 11. Behavioral Abnormalities in Auts2 Mutant Mice

Behavioral studies have been conducted using several different types of *Auts2* mutant mice. Homozygous pups with neuron-specific deletion of *Auts2* exhibit defects in motor abilities, such as geotaxis and righting reflex [27]. Adult homozygotes with cerebellar ablation of *Auts2* also showed impaired balance control and motor learning [34]. Moreover, *Auts2* heterozygous conventional KO mice exhibit autistic-like behaviors, such as social deficits and altered vocal communication [25,68]. Intriguingly, loss of *Auts2* not only reduces the number of ultrasonic vocalizations (USVs) but also the complexity of USV syllables emitted from male mice during courtship behaviors [25]. Defects in social behaviors and vocal communication are well recapitulated by selective deletion of *Auts2* in excitatory neurons in the adult forebrain [25], implying that the synaptic regulation by AUTS2 significantly contributes to social behaviors in mice. Furthermore, *Auts2* mutant mice manifest altered anxiety as well as abnormal startle responses to auditory and nociceptive stimuli [25,68], which may be due to the disturbed E/I balance in sensory cortices. Both phenotypes are often observed in individuals with ASD, in addition to other impairments [3]. In addition, loss of *Auts2* results in defects in cognitive and memory function, suggesting that AUTS2 contributes to neurocognitive brain functions.

## 12. Conclusions and Perspectives

Over the last few decades, multiple mutations within the *AUTS2* locus have been identified in patients with NDDs, identifying it as a causative factor for neurodevelopmental and psychological disorders such as ASD, ID, ADHD, and schizophrenia. Accumulating evidence has revealed that AUTS2 is a key regulator involved in a wide range of neurodevelopmental processes, from prenatal neurogenesis to the assembly of neural circuits in mature brains. In cell nuclei, AUTS2 participates in the regulation of gene expression through epigenetic modulation. Cytoplasmic AUTS2 contributes to the organization of cytoskeletal rearrangements to regulate prenatal cortical development, including neuronal migration and neurite morphogenesis. Postnatally, AUTS2 modulates the E/I balance of synaptic inputs by restricting the number of excitatory synapses.

Nevertheless, some key issues remain to be addressed. Although several lines of evidence have suggested that multiple types of AUTS2 isoforms are potentially expressed in various brain regions, the exact mechanisms by which their expression is regulated and their precise spatiotemporal organization has not been analyzed. In addition, the biological function of the individual AUTS2 isoforms is not well understood, and how the loss- or gain-of-function of these isoforms affects neurodevelopment as well as behaviors remains unknown. Moreover, the specific AUTS2 downstream target genes and molecular pathways involved in neurodevelopment, including neurogenesis and synaptogenesis, have not been well determined. Further elucidation of the molecular function of AUTS2, as well as a more comprehensive understanding of the AUTS2-mediated gene expression network pathways in neural development will help to unravel the complex pathological mechanisms of NDDs and provide promising insights for new therapeutic strategies to treat these disorders.

## Data Availability

Not applicable.
